# Human Gut Bacteriophageome: Insights Into Drug Resistance Mechanisms in Tuberculosis

**DOI:** 10.1155/ipid/8811027

**Published:** 2025-06-16

**Authors:** Erfaneh Jafari, Reza Azizian, Mohsen Tabasi, Morteza Banakar, Kamran Bagheri Lankarani

**Affiliations:** ^1^Pediatric Infectious Diseases Research Center (PIDRC), Tehran University of Medical Sciences, Tehran 14197, Iran; ^2^Biomedical Innovation and Start-Up Student Association (Biomino), Tehran University of Medical Sciences, Tehran 14317, Iran; ^3^Center for Inflammation and Lung Research, Temple University, Philadelphia 19140, Pennsylvania, USA; ^4^Health Policy Research Center, Shiraz University of Medical Sciences, Shiraz 71348, Fars, Iran; ^5^Dental Research Center, Tehran University of Medical Sciences, Tehran 14399, Iran

**Keywords:** drug resistance, microbiota, phageome, tuberculosis

## Abstract

Tuberculosis (TB), caused by *Mycobacterium tuberculosis*, remains a major global health burden. The emergence of drug-resistant strains presents a critical challenge in TB management. The recent research has explored the interaction between TB and the human gut bacteriophage community (phageome). The gut phageome plays a crucial role in regulating microbial diversity and functionality, and its composition and function have been linked to various health conditions. Examining the gut phageome through metagenomic analysis provides insights into its composition, role in health, and interactions with the host immune system. Exploring the interaction between the gut phageome and *M. tuberculosis* may reveal how phages affect the bacterium's pathogenicity, survival, and mechanisms of drug resistance. Understanding the gut phageome's impact on TB drug resistance could inform novel therapeutic strategies, such as phage therapy, and highlight the importance of microbiome-based interventions in combating drug-resistant TB strains. This review explores the role of the gut phageome in influencing drug resistance in TB, focusing on interaction mechanisms and potential therapeutic implications, synthesizing current research findings, and identifying knowledge gaps in this emerging field. This review also synthesizes the current evidence on the gut phageome's role in TB drug resistance, focusing on phage-mediated horizontal gene transfer (e.g., rpoB, katG), immune modulation, and preclinical efficacy of mycobacteriophage therapies. Key findings highlight phage cocktails (e.g., DS6A, D29 LysB) as promising adjuncts to antibiotics, reducing *M. tuberculosis* burden in murine models. These insights advocate for phage therapy as a complementary strategy against drug-resistant TB, urging clinical validation to bridge the existing knowledge gaps.

## 1. Background

Tuberculosis (TB), caused by *Mycobacterium tuberculosis* (Mtu), is a contagious disease primarily affecting the lungs but capable of spreading to other organs. TB remains a significant global health issue, with over 10 million new cases and 1.3 million deaths annually, particularly in low-income and resource-limited areas [1]. The impact of TB extends beyond its health implications, as it also contributes to social and economic burdens in affected communities [2]. Therefore, drug resistance in TB is a pressing concern that complicates treatment and control efforts. Drug-resistant strains mainly arise from the improper use of antibiotics, incomplete treatment courses, and inadequate healthcare infrastructure [3]. Although advances have been made in TB diagnostics and treatment, the management of drug-resistant TB continues to pose significant challenges, underscoring the pressing need for novel approaches to combat this issue [1, 4].

Introducing the concept of gut phageome sheds light on the complicated relationship between humans and their gut microbiota. The phageome includes all the virome (phageome) in the gut, which regulates microbial diversity and functionality [5]. Research has shown that gut phageome composition changes can impact host health, immunity, and susceptibility to various diseases, including infectious conditions like TB [6, 7]. Recent studies have investigated how the human gut microbiome, specifically the gut bacteriophage community (phageome), may impact TB development and drug resistance. Despite the promising insights provided by these studies, it is important to note their limitations. For example, the impact of phages on TB drug resistance has often been inferred from correlational data rather than direct mechanistic studies [8]. Moreover, the variability in gut phageome composition across individuals and over time poses a challenge for establishing consistent therapeutic strategies [9].

Exploring the potential connection between drug resistance in TB and the phageome is a fascinating area of study, as it may provide novel insights into the complex interplay between the host, microbiome, and pathogen. Understanding these interactions could lead to the development of innovative strategies for combating drug-resistant TB strains. The gut virome, including phages, may influence the immune response and microbial interactions within the host, potentially affecting the pathogenesis of TB and the advancement of drug resistance [10, 11].

TB drug resistance arises from mutations in critical genes (e.g., *rpoB* for rifampicin, *katG* for isoniazid) and adaptive mechanisms like efflux pumps. Current therapies struggle with prolonged treatment durations and toxicity, underscoring the need for novel approaches. The gut phageome's role in modulating bacterial resistance remains underexplored, particularly its capacity to transfer resistance genes or alter Mtu pathogenicity. Exploring the interaction between the gut phageome and Mtu may reveal how phages affect the bacterium's pathogenicity, survival, and mechanisms of drug resistance. Although these interactions are largely speculative at this stage, they are supported by initial evidence, suggesting that phages can influence bacterial gene expression and immune modulation [12, 13].

This review aims to elucidate the role of gut phageome in influencing drug resistance in TB, exploring potential therapeutic implications and research gaps in this emerging field. Understanding the intersection of TB, drug resistance, and the gut phageome represents a complex yet promising edge in infectious disease research (Table 1).

## 2. The Human Gut Phageome

### 2.1. Overview of the Gut Phageome

The human gut is a complex ecosystem containing various microorganisms, including bacteria, archaea, fungi, and viruses. Bacteriophages, or phages, are the most prevalent and diverse viruses in the gut [9]. Phages are bacterial viruses that infect and reproduce inside their host bacteria, significantly influencing the structure and function of the gut microbiome [22].

### 2.2. Definition and Composition

The gut phageome is the total genetic content of all phages in the human gut. It encompasses a wide variety of phage types, including both lytic and temperate phages. Lytic phages follow a virulent lifecycle: They infect, replicate within, and lyse their bacterial hosts, releasing new phage particles. In contrast, temperate phages can incorporate their genetic material into the bacterial genome, creating inactive prophages. These prophages can later be activated to initiate the lytic cycle [8].

The makeup of the gut phageome is unique and constantly changing, differing among people and fluctuating over time. Age, nutrition, geography, and health might impact the kind and quantity of phages in the gut [9]. Studies have shown that the gut phageome is dominated by double-stranded DNA (dsDNA) phages belonging to the order *Caudovirales*, which includes the families *Myoviridae, Siphoviridae*, and *Podoviridae* [23, 24]. Studying the gut phageome's makeup is essential for investigating its impact on health and illness, as phage variety and abundance alterations have been linked to different pathological states. By characterizing the gut phageome, researchers can gain insights into the complex interactions between phages, bacteria, and the host immune system.

### 2.3. Methods for Studying the Gut Phageome

Metagenomic analysis has emerged as a powerful tool for studying the gut phageome. This method entails sequencing the whole DNA isolated from fecal samples without relying on culture-based techniques [25]. Metagenomic sequencing enables identifying and characterizing familiar and new phages in the gut [24]. For instance, similar metagenomic approaches have been effectively utilized to study oral bacteriophages, providing insights into their interactions with the oral microbiota and their implications for health and disease [26].

In studying the gut phageome, two primary metagenomic strategies are utilized: shotgun metagenomics and viral metagenomics (viromics) [25]. Shotgun metagenomics sequences the total DNA from a sample, capturing bacterial and viral sequences. Computational tools then differentiate phage sequences from bacterial ones [27]. Conversely, viromics enriches the viral fraction before sequencing through methods like filtration, density gradient centrifugation, and nuclease treatment to eliminate bacterial cells and free nucleic acids [28].

Once the phage sequences are obtained, bioinformatic tools are used for assembly, annotation, and taxonomic classification [28]. Reference databases such as RefSeq, a comprehensive database of annotated genomic, transcript, and protein sequences, and the Gut Phage Database (GPD) [29], a curated database of phages found in the human gut, aid in identifying and characterizing known phages. However, the bulk of gut phages are poorly understood, making it difficult to identify and assign functions to them [22].

Despite its utility, metagenomics faces challenges, including incomplete reference databases (e.g., underrepresentation of mycobacteriophages in the GPD) and difficulty distinguishing prophages from lytic phases. Additionally, viromics enrichment steps may bias against low-abundance phages, complicating the detection of novel Mtu-targeting phages.

### 2.4. Role in Human Health and Disease

#### 2.4.1. Gut Homeostasis and Immune Responses

The gut phageome significantly regulates the makeup and function of the gut microbiome, impacting human health and disease [9]. Phages influence bacterial communities through host cell lysis, horizontal gene transfer (HGT), and modulation of bacterial metabolism [30]. The lysis of bacterial hosts releases components such as lipopolysaccharides (LPS) and peptidoglycan, potentially triggering immune responses and influencing gut homeostasis [31].

#### 2.4.2. Association With Diseases

Changes in the gut phageome have been linked to various disorders, including inflammatory bowel disease (IBD) [17], *Clostridium difficile* infection (CDI) [32], and Type 2 diabetes (T2D) [33]. For instance, studies have shown an increased abundance of Caudovirales phages in the gut of IBD patients compared to healthy controls [34]. Similarly, a dysbiotic gut phageome has been observed in CDI patients, characterized by a higher prevalence of temperate phages and a lower diversity of lytic phages than in healthy individuals [34]. These changes in the gut phageome are associated with modifications in bacterial composition and immunological function, indicating their potential involvement in disease development.

#### 2.4.3. Antibiotic Resistance

The gut phageome has also been implicated in the emergence and spread of antibiotic resistance in bacterial pathogens, including Mtu. Phages can encode antibiotic resistance genes and transfer them to their bacterial hosts through transduction, contributing to the development of drug-resistant strains [35]. In TB, phages have been shown to mediate the transfer of resistance genes, highlighting their role in the evolution of drug-resistant Mtu [36].

### 2.5. Gut Phageome Interaction With the Host

The gut phageome does not passively exist in the human gut but instead actively engages with the host immune system and impacts the makeup and operation of the gut microbiota [31, 37]. Phages can activate the immune system by interacting with phage-associated molecular patterns (PAMPs) recognized by host pattern recognition receptors (PRRs) [37]. This connection can trigger innate and adaptive immune responses, influencing the host's immunological environment [38].

#### 2.5.1. Mechanisms of Interaction

Phages engage with the host immune system through many methods, including direct recognition by PRRs, modulation of bacterial antigens, and translocation across the gut barrier [39]. Toll-like receptors (TLRs), particularly TLR9, have been shown to recognize phage DNA and trigger the production of pro-inflammatory cytokines such as Type I interferons (IFNs) [37]. Recognition of phage PAMPs can trigger innate immune cells, including dendritic cells and macrophages, influencing the adaptive immune response [38].

Phages can also indirectly influence the host immune system by modulating the expression of bacterial antigens and virulence factors. Lysogenic phages can modify the expression of bacterial surface proteins by integrating their genetic material into the bacterial genome, thereby influencing how they are recognized by the immune system [19]. Additionally, phage-mediated lysis of bacterial cells can release bacterial components, such as LPS and peptidoglycan, which can stimulate immune responses and influence gut homeostasis [31].

Recent research has demonstrated that phages can cross the gastrointestinal barrier and spread to other organs, such as the liver, spleen, and lungs [40]. The movement of phages can impact the host's immune system, perhaps leading to the onset of autoimmune diseases and persistent inflammation [31]. The mechanisms underlying phage translocation and their impact on host immunity are poorly understood and require further investigation.

#### 2.5.2. Impact on Host Immunity and Gut Microbiota

The relationship between the gut phageome and the host immune system significantly affects human health and illness [41]. Phages can alter the structure and activity of the gut microbiota by infecting and breaking down specific bacterial hosts [42]. This selective pressure can lead to shifts in the relative abundance of bacterial species, altering the ecological balance of the gut microbiome [9].

**Figure 1:**
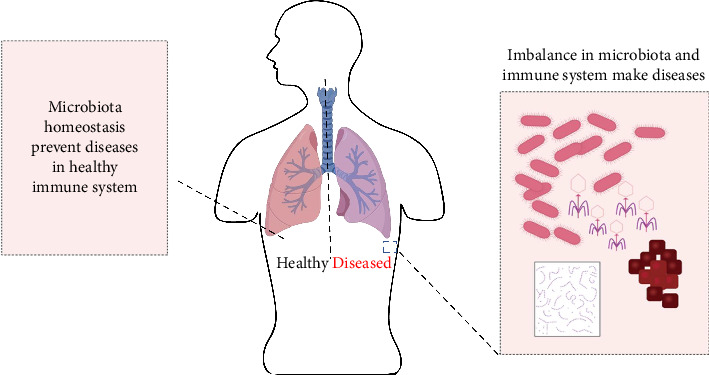
The microbial communities are symbiosis with the host, contributing to homeostasis and regulating immune function. However, microbiota dysbiosis can lead to dysregulation of bodily functions and diseases such as IBD, Type 2 diabetes, and respiratory diseases.

Alterations in the gut microbiota caused by phages can greatly impact the host's immune response and illness vulnerability [43]. For example, the depletion of certain bacterial species by phages can create ecological niches that favor the growth of opportunistic pathogens, leading to dysbiosis and inflammation [42]. Phages may help maintain a healthy gut microbiota by inhibiting the excessive development of harmful bacteria and facilitating the establishment of helpful species [44] (Figure 1).

The gut phageome's influence on host immunity goes beyond the gut environment, affecting systemic immunological responses and the onset of immune-mediated diseases [31]. Phage-induced inflammation and immunological activation are linked to the development of several disorders, such as IBD [43], Type 1 diabetes [45], and multiple sclerosis [46]. However, the specific interactions between the gut phageome, host immunity, and Mtu infection are still largely unexplored and warrant further research [12].

## 3. Mtu and the Gut Phageome

Mycobacterium tuberculosis is the causative agent of TB. It is a highly effective pathogen that has evolved various strategies to evade the host immune system and establish a persistent infection. While the lungs are the primary site of Mtu infection, recent research suggests that the gut may also play a role in TB development [47]. The gut phageome, with its diverse collection of bacteriophages, may influence the survival and pathogenicity of Mtu through various mechanisms [46].

### 3.1. Characteristics of Mtu Relevant to the Gut Phageome

Mtu is an acid-fast bacillus with a unique cell wall structure that contributes to its resilience and ability to persist within host cells. The cell wall of Mtu is rich in lipids, including mycolic acids, which form a hydrophobic barrier that protects the bacterium from environmental stressors and immune responses [47]. These lipids also serve as potential targets for phage adsorption and infection, as some mycobacteriophages have been shown to recognize and bind to specific lipid moieties on the Mtu cell surface [36].

Mtu has a slow growth rate and the ability to enter a dormant state, which allows it to persist in the host for extended periods. This dormancy is defined by decreased metabolic activity and heightened tolerance to drugs and immunological agents [48]. The gut phageome may influence the establishment and maintenance of Mtu dormancy through mechanisms such as phage-mediated HGT and modulation of host immune responses [12].

### 3.2. Evidence for Interactions Between the Gut Phageome and Mtu

Recent studies have provided evidence for the presence of Mtu in the gut and its potential interactions with the gut microbiota and phageome [47]. In a study by Luo et al., Mtu was detected in the fecal samples of TB patients, suggesting that the gut may serve as an extrapulmonary reservoir for the pathogen. The presence of Mtu in the gut raises the possibility of its interaction with the resident phageome and the potential impact on TB pathogenesis [49].

Phages have been shown to infect and lyse Mtu, indicating their potential role in shaping the population dynamics of the pathogen [47]. Mycobacteriophages, phages specific to mycobacteria, have been extensively studied and have provided valuable insights into the biology and genetics of Mtu. The isolation of mycobacteriophages from the gut phageome suggests that they may influence the survival and persistence of Mtu in the gut environment [12].

In addition to direct interactions, the gut phageome may indirectly influence Mtu through its effects on the gut microbiota [47]. Phage-mediated alterations in the composition and function of the gut microbiota could create ecological niches that favor the growth and survival of Mtu. For example, phage-induced lysis of certain bacterial species may release nutrients and reduce competition for resources, thereby promoting the growth of Mtu [9, 12].

### 3.3. Influence of the Gut Phageome on the Pathogenicity and Survival of Mtu

The gut phageome can influence the pathogenicity and survival of Mtu through various mechanisms. These include HGT, modulation of host immune responses, and alteration of the gut microbiota composition [12].

#### 3.3.1. Modulation of Bacterial Competition

Phages can facilitate the transfer of virulence factors and antibiotic resistance genes to Mtu, enhancing its pathogenicity and ability to evade host defenses [50]. The acquisition of phage-encoded virulence factors, such as toxins and adhesins, may increase the invasiveness and persistence of Mtu in the gut and other extrapulmonary sites [13].

Phages play a crucial role in regulating bacterial populations within the gut. By infecting and lysing specific bacterial hosts, phages can alter the competitive dynamics among bacterial species. This modulation can impact the survival and growth of Mtu in the gut environment. For instance, certain phages might reduce the abundance of bacterial species competing with Mtu, thereby influencing its pathogenicity [51, 52].

#### 3.3.2. Horizontal Gene Transfer

Phages can mediate HGT, a process that allows the spread of genetic material, including antibiotic resistance genes, among bacterial populations. This transfer can occur through transduction, where phages package and transfer bacterial DNA to new bacterial hosts. In the context of Mtu, phage-mediated gene transfer could contribute to the dissemination of drug resistance genes, complicating treatment efforts [53, 54]. For instance, phages like DS6A have been shown to transduce katG mutations, conferring isoniazid resistance in Mtu. Similarly, phage-mediated transfer of rpoB variants may contribute to rifampicin resistance, highlighting phages as vectors for resistance gene dissemination [55].

#### 3.3.3. Impact on Host Immune Response

Phages can directly modulate host immune responses, potentially creating a favorable environment for Mtu survival and dissemination. Phage-induced inflammation and immune activation may disrupt the gut barrier, facilitating the translocation of Mtu to other organs [31]. Phages can also affect the host immune response by lysing bacterial cells and releasing bacterial components that interact with the immune system. This interaction can lead to changes in the immune landscape of the gut, potentially influencing the host's ability to mount an effective immune response against Mtu. For example, releasing bacterial antigens upon phage-induced lysis might enhance immune recognition and response to Mtu, affecting its survival and pathogenicity [24].

Furthermore, phages can indirectly influence the host's ability to mount an effective immune response against Mtu. This can occur through mechanisms such as inducing immunosuppressive cytokines or inhibiting the activation of phagocytic cells [37].

#### 3.3.4. Alteration of Gut Microbiota Composition

The gut phageome may further influence Mtu pathogenicity by altering the composition and function of the gut microbiota [47]. Phage-mediated dysbiosis can lead to the depletion of beneficial bacteria and the expansion of opportunistic pathogens, creating a favorable niche for Mtu colonization and growth [9]. Dysbiosis-induced inflammation and impaired gut barrier function may also facilitate the dissemination of Mtu from the gut to other organs, contributing to the development of extrapulmonary TB [47].

**Figure 2:**
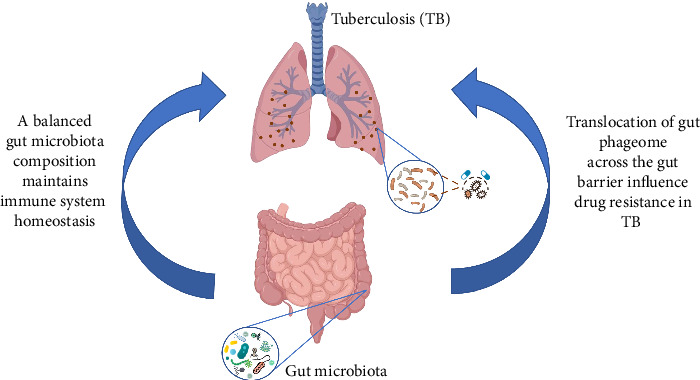
Phage–drug interactions with *M. tuberculosis* are well-documented, demonstrating the potential for phage therapy to treat drug-resistant tuberculosis (DRTB) strains.

Understanding these mechanisms is crucial for developing novel therapeutic strategies (Figure 2). By modulating the gut phageome or leveraging phage therapy, it may be possible to alter the interactions between Mtu, the gut microbiota, and the host immune system, thereby influencing the course of TB infection and its resistance to treatment.

Phage-induced lysis of Bacteroides spp. increases mucosal oxygen levels, favoring Mtu survival via the DosR dormancy regulon [56]. Additionally, phage-mediated suppression of *Faecalibacterium prausnitzii* reduces anti-inflammatory butyrate production, exacerbating gut barrier dysfunction and systemic inflammation [57].

## 4. TB and Drug Resistance

### 4.1. Mechanisms of Drug Resistance in TB

Drug resistance in TB arises from complex processes that reduce treatment efficacy. Mtu, the causative agent of TB, acquires resistance mainly through genetic mutations and adaptive responses [58]. Genetic mutations in key genes can alter drug-targeted proteins, reducing antibiotic effectiveness. For instance, *rpoB* gene mutations lead to rifampicin resistance, while *katG* and *inhA* gene mutations cause isoniazid resistance [59, 60]. Adaptive responses allow the bacterium to modify its physiological functions in response to drug exposure, further developing resistance [4, 58]. Understanding these mechanisms is crucial for developing innovative therapeutic strategies to combat drug-resistant TB strains.

### 4.2. Current Challenges in TB Drug Resistance

Despite advancements in TB management, the prevalence of drug-resistant strains poses significant challenges to global health efforts. Existing treatments for TB are limited by factors such as prolonged duration, potential side effects, and the necessity for multidrug therapy [61]. Multi–drug-resistant (MDR) and extensively drug-resistant (XDR) TB strains complicate treatment results. MDR-TB is resistant to the two most effective first-line medications, whereas XDR-TB shows resistance to additional second-line treatments [61]. The rise in medication resistance highlights the immediate requirement for new treatments and improved surveillance methods to control the spread of drug-resistant TB strains.

To effectively address the mechanisms and challenges of drug resistance in TB, a multifaceted approach is necessary, integrating research, innovation, and global collaboration [62]. By clarifying the genetic foundations of drug resistance and exploring adaptive responses in Mtu, scientists can unravel new targets for drug development and precision medicine interventions. Overcoming the limitations of existing TB treatments necessitates the expansion of new antimicrobial agents, rapid diagnostics, and optimized treatment protocols tailored to drug-resistant strains.

## 5. Phageome's Role in TB Drug Resistance

### 5.1. Direct Interactions

The relationship between the phageome and drug resistance in TB provides novel insights for combating antimicrobial resistance. Phages, viruses that infect bacteria, play a crucial role in shaping Mtu drug resistance dynamics [18]. Documented direct interactions between phages and Mtu highlight the potential of phage therapy as a promising treatment for drug-resistant TB strains [20, 63]. By targeting specific bacterial species, phages can disrupt the growth and survival of Mtu, offering a personalized approach to combating resistant strains.

### 5.2. Indirect Interactions

In addition to direct interactions, the phageome's influence extends to indirect interactions that control the host's immunity and microbial ecosystems. Studies have revealed the impact of phages on gut microbiota composition, which can have consequential effects on TB susceptibility and treatment outcomes [50, 64]. By shaping the microbial balance within the gut, phages can alter the host's immune response to Mtu infection, potentially enhancing or diminishing the effectiveness of existing drug regimens [20, 65]. This complex interplay highlights the significance of considering the full ecosystem of microbial interactions in the context of TB drug resistance.

The prospect of leveraging phage therapy as a targeted approach for drug-resistant TB holds significant promise in the realm of precision medicine. By exploiting the specificity of phages for Mtu, researchers aim to develop tailored cocktails that can effectively combat resistant strains while minimizing collateral damage to the host's microbiota [50, 66]. This personalized treatment strategy offers a viable alternative to traditional antibiotic therapies, which often face challenges in addressing drug resistance and off-target effects.

Moreover, the modulation of immune responses by phages presents a unique opportunity to enhance the host's defense mechanisms against TB infection [67]. By adjusting the immune system's recognition and clearance of Mtu, phages can synergize with conventional drug treatments to improve therapeutic outcomes and reduce the risk of resistance emergence [68]. Embracing the multifaceted role of the phageome in TB drug resistance opens avenues for innovative interventions that are personalized, effective, and sustainable in the fight against resistant TB strains.

## 6. Therapeutic Implications

### 6.1. Current Research on Phage Therapy for TB

Treating nontuberculous mycobacterium (NTM) infections is challenging due to the need for prolonged antibiotic treatments that have poor effectiveness. Mycobacteriophages, which are viruses that infect Mycobacterium hosts, have potential as therapeutic agents. Many patients reported favorable results in 20 compassionate use cases, with no signs of phage resistance, even when a single phage was delivered. The therapeutic efficacy of phages is now restricted by the variability in phage susceptibility and a limited selection of beneficial phages [69, 70]. While most compassionate use cases have focused on *M. abscessus* infections, other Mycobacterium pathogens, such as *Mycobacterium avium complex* (MAC) and Mtu, require alternative therapies [71].

Phages have shown promise in treating some of these infections, but phage susceptibility varies greatly, necessitating personalized treatments. A group of 20 patients received phage therapy compassionately, supporting the need for future assessment of phages in Mycobacterium infections. Over half of the patients had positive responses, with several infections completely resolved and one undergoing successful lung transplantation. Some individuals saw minimal clinical improvement, and the reason for this variation in response is unknown [69].

In the case of Mtu, which is treatable but often noncompliant and prone to antibiotic resistance, phages could potentially shorten treatment, enhance compliance, and reduce resistance. Clinical trials could provide insights into the prospects for phage therapy. However, phages' ability to access intracellular bacteria and the complexities of the disease should be considered.

Phage DS6A demonstrates a reduction in Mtu levels within various organs of mice, with a more significant impact observed in the spleen. These results highlight the potential of developing phage therapy as an effective treatment option against Mtu infections [15].

Notably, mycobacteriophage D29 LysB shows promise as a candidate for enhancing the current anti-infection regimen, particularly when used in conjunction with isoniazid and rifampicin. While the in vitro studies reveal an additional antibacterial effect of LysB when combined with existing medications, further research is needed to confirm its efficacy in animal models. Nevertheless, these findings provide proof of concept regarding the antimicrobial activity of LysB against Mtu [16].

In the domain of genetic manipulation, mycobacteriophages have shown potential in enhancing traits in mycobacteria. To leverage bacteriophages as a therapeutic strategy, a comprehensive understanding of their influence on bacterial populations is essential. Utilizing computer simulations can offer valuable insights into specific interactions between mycobacteriophages and their hosts. Simulation models enable a deeper comprehension of system behavior under varying parameters, aiding in the prediction and understanding of phage–host interactions [21].

Clinical isolates of Mtu are more homogeneous than those of *M. abscessus* and other NTM strains, and the phage infection profile is much less variable. A relatively small phage cocktail (3–5 phages) infects multiple isolates of the major Mtu lineage [63].

It is unlikely that personalization will occur, which is significant for a pathogen that takes so long to develop. Clinical trials testing antimycobacterial activity can be expected and may provide insight into treatment prospects. Overall, phage therapy shows potential for treating NTM infections, but further research, clinical trials, and improvements in phage susceptibility are needed to harness their therapeutic benefits [63, 72].

While using phages to overcome bacterial defense mechanisms holds promise, some potential limitations and challenges must be addressed. One concern is the development of phage resistance, as bacteria can evolve mechanisms to evade phage infection, such as altering cell surface receptors or activating antiviral defense systems [73]. To address this issue, researchers are investigating the utilization of phage cocktails, including several phages with diverse host ranges and modes of action [74]. Additionally, the continuous evolution of phage cocktails may be necessary to keep pace with the evolving bacterial resistance landscape.

Although there is a high demand for new treatments, the compassionate use of Mtu infections is uncommon due to the frequent discovery of new antibiotics often evaluated for treatment during the early stages of approval. Optimism should be cautious because the absence of clinical data supports the idea that phages may attack intracellular bacteria. However, decreasing the extracellular bacterial population using phages and antibiotics is feasible, allowing for further research [72].

*Mycobacterium smegmatis*, a nonpathogenic relative of Mtu, has been pivotal in mycobacteriophage research. Its fast growth and genetic tractability enable high-throughput phage isolation and characterization, providing insights into phage–host interactions applicable to TB [75, 76].

### 6.2. Potential for Phages to Combat Drug Resistance

Bacteria can alter their receptors to evade phage infections through a process known as phase variation. Bacteria can create an extracellular matrix to serve as a barrier against phages. However, specific phages can bypass this defense by using enzymes that break down the matrix [77, 78]. Bacteria can also resist phage infections through superinfection exclusion systems and restriction–modification enzymes. Another defensive strategy is the synthesis of secondary metabolites that prevent phage infection [79, 80].

Universities and governments are funding bacteriophage-based treatments and supporting trials to expedite the use of phage therapy in medical facilities. Securing patents for phages can be difficult, yet modified phages are increasingly recognized for their medicinal benefits [81, 82]. Modified bacteriophages can transport harmful genes and expand the types of organisms they can infect. Yet, uncertainties persist about manipulating phages, including regulating lysis duration and selecting the most effective therapy for illnesses with substantial bacterial populations. Initiatives such as “Sea-Phages” have aided in uncovering new phages and establishing phage collections; quicker isolation techniques are required to address evolving resistance infections. In the future, phage treatment has the potential to be a flexible method for treating bacterial illnesses with careful planning and data analysis [83].

Unlike other infections, using phages to manage Mtu is advantageous due to the minimal variance in phage susceptibility across clinical isolates. Research indicates that a cocktail consisting of just five phages may be suitable for clinical studies to assess phage effectiveness and safety. This method reduces resistance issues and removes the necessity of screening patient isolates in advance [63]. Before clinical examination, the five-phage cocktail may be improved by adding more phages and eliminating integration cassettes. Testing a wider variety of clinical isolates, drug-resistant strains, and diverse lineages of Mtu is crucial. The extensive coverage offered by the phages, particularly across various strains, indicates that the majority of Mtu strains might be susceptible to infection and elimination by some of the phages in the cocktail [84]. The phage “space” for TB therapy differs from other bacterial diseases, as lytic *Myoviruses* and *Podoviruses* are frequently utilized for other pathogens.

**Figure 3:**
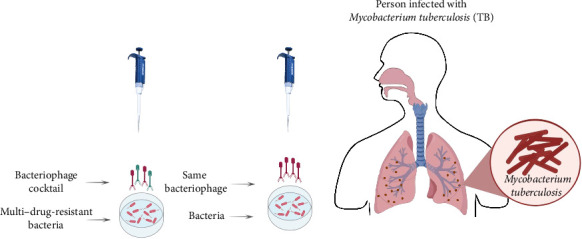
Cocktail phages are determined by their effective potential against specific multidrug-resistant bacteria. This therapy for multidrug-resistant bacteria is more applicable than homologs or unique phage therapy.

By eliminating the repressor gene, engineering techniques can turn temperate phages into lytic ones (Figure 3). Recombineering tools are utilized to remove integrase genes in approaches such as BRED and CRISPR [85, 86]. Identifying phages that effectively infect and eliminate Mtu strains with low resistance rates and are compatible with antibiotics has cleared the way for clinical assessment of bacteriophages for treating TB. Phages have a favorable safety profile in humans, indicating their potential for therapeutic use [50, 86].

## 7. Gut Microbiota Manipulation

### 7.1. Phage Therapy for Altering the Gut Phageome

Phage therapy has emerged as a promising approach to modulating the gut phageome and restoring microbial balance in the context of IBD [37]. IBD, which includes Crohn's disease and ulcerative colitis, is characterized by chronic inflammation of the gastrointestinal tract and has been linked to gut microbiome dysbiosis [87]. Microbiota-targeted treatments, such as probiotics and fecal microbiota transplants, have demonstrated promise in addressing immune system abnormalities [88, 89]. By targeting specific bacterial species associated with IBD pathogenesis, phage therapy aims to reduce inflammation and promote the growth of beneficial bacteria [90].

Recent studies have demonstrated the potential of phage therapy in alleviating IBD symptoms and modulating the gut microbiome [37, 91]. In IBD treatment, phage therapy mainly targets adherent invasive *Escherichia coli* (AIEC), which is more prevalent in IBD patients. In addition to animal models, in vitro models are also used to study the therapeutic activity of gut phages. An intestinal epithelium model has been developed to understand phage–bacteria interactions and the protective effects of phage therapy [92].

Research on the effectiveness of phage therapy in treating IBD is still limited. It is essential to comprehend the significance of gut phages in developing IBD and T2D. Additional experimental data are needed to understand how phages impact IBD by influencing bacterial activity and immune response [93]. While the findings discussed here are promising, further research is required to elucidate the capacity of phages to regulate the balance of gut microbiota and immune response. Challenges such as phage specificity, potential immune responses, and the development of phage resistance will need to be addressed to optimize therapeutic outcomes. Also, addressing safety and administration time difficulties is crucial [94, 95].

### 7.2. Fecal Virome Transplantation (FVT) for Altering Gut Phageome

FVT has been utilized in conditions such as obesity, demonstrating its potential to modify the gut microbiome for therapeutic purposes [14, 96]. FVT involves transferring viral components from a healthy donor's stool to a recipient's gut to restore the microbiome composition. On the other hand, phage therapy targets specific bacterial strains causing infections by isolating and transplanting effective phages [97, 98].

**Figure 4:**
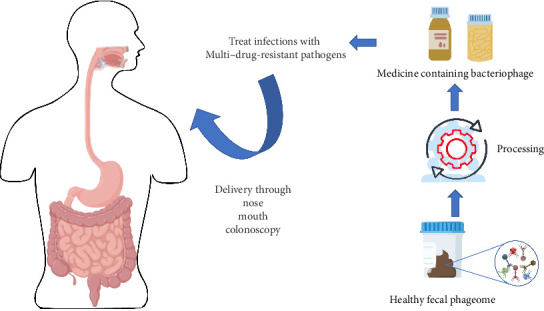
Fecal microbiota transplantation (FMT) has shown promising results in restoring the gut microbial balance and modulating immune responses in IBD patients. Given the similarities in immunomodulation and dysbiosis between IBD and TB, this review hypothesized that FMT could also provide therapeutic benefits as an adjunct treatment in TB (exploring the potential of fecal microbiota transplantation as a therapy in tuberculosis and inflammatory bowel disease; Adrian Boicean).

FVT specifically focuses on the virome, which includes viruses and phages, rather than a wide range of microorganisms like fecal microbiota transplantation (FMT). The donor's fecal matter is processed to eliminate whole bacterial cells before being sent to the recipient to alter bacterial populations. FVT reduces the hazards linked to fecal microbiota transplantation (FMT), including bacterial infections [99, 100] (Figure 4).

Preclinical studies have shown promising results of FVT in obese mice, including weight reduction and improvements in glucose tolerance and liver pathology. FMTs, which involve transplanting bacteria, viruses, and phages, have been used in humans for conditions like CDI, IBD, and autism spectrum disorder. Changes in the phageome, the community of phages in the gut, have been observed post-FMT and correlated with clinical outcomes [101, 102]. However, there are challenges associated with FVT. The long-term effects and lack of studies in this area need further investigation. There is also a risk of transmitting unwanted viruses from the donor to the recipient. Thorough screening of the donor virome and modification of the filtrate could minimize this risk.

Phage treatment specifically targets certain germs while reducing unintended consequences. It is a potential antibiotic substitute since it has a more specific target range and fewer negative effects in human hosts. Studies confirm that phage treatment is effective and safe in treating infectious disorders caused by bacterial strains resistant to many drugs. Phage treatment often involves creating a phage cocktail. Challenges are deciding the distribution method, dose, stability of phage formulations, and ethical considerations [103–107].

### 7.3. Challenges and Future Directions

The use of mycobacteriophages in TB control has significant clinical potential in several areas, including diagnosis, transmission interruption, and treatment. Specific phage candidates need to be identified, resistance mechanisms studied, and infection variations determined among different strains and genetic lineages. Phages may not be effective for directly managing TB infections due to the intricacy of internal infections, but they might target extracellular bacteria in later stages. However, in vitro susceptibilities may not correlate with phage infection profiles in infected individuals, necessitating clinical trials or evaluation in nonhuman primates [36].

Compared to other pathogens, Mtu clinical isolates show relatively slight variation in phage susceptibility. Genomic research has broadened this knowledge and indicates that a combination of just five phages might be effective and safe in human trials. The cocktail may be used without having to prescreen patient isolates for phage susceptibility, a difficult and time-consuming process with slow-growing bacteria [84].

Further refinement of the five-phage cocktail is expected before clinical evaluation, with the potential inclusion of other phages that infect and kill a broader range of strains. Limitations include the integration cassette in one phage, the need to amplify and propagate phages on slow-growing MTBC strains, and the less effective performance of certain phages [108].

While the tested phages and cocktail were effective against most strains, lineage 6 presented some susceptibility challenges. Clinical trials may need to consider the prevalence of lineage six strains in specific regions [109, 110]. Other lineages, such as L7, L8, and L9, have not been tested extensively, and it would be beneficial to examine more clinical isolates and drug-resistant strains from lineages L2 and L4 [109, 110].

TB treatment phages are primarily lytic descendants of temperate parent phages, setting them apart from phages employed against other bacterial diseases. Engineering techniques can transform temperate phages into lytic phages by eliminating the repressor gene, with the additional recommendation of deleting the integrase genes. Clinical assessment of bacteriophages for TB treatment is now possible due to a collection of phages that effectively target and eliminate a wide variety of Mtu strains in conjunction with antibiotics. The safety profiles of phages in humans are exceptional, enabling the investigation of their extensive use in treatment [111].

Multiple studies have indicated that most viral populations cannot be classified into a specific taxonomic group, so they are called viral “dark matter.” Finkbeiner et al. utilized a new “micro-mass sequencing” method to identify recognized and unidentified viruses in samples from individuals with diarrhea [112]. Their research demonstrates a strong correlation between the identified viruses and gastrointestinal well-being. Studying these viral genomes can provide a deeper insight into the origins and therapies of prevalent illnesses. Several studies have researched methods to analyze this elusive viral genetic material [113]. Benler et al. identified 3738 complete phage genomes from 451 species by whole gut metagenomics, uncovering several unidentified human gut phages [114]. In the future, new research and methodologies are required to simplify the analysis of these intricate intestinal phageomes.

Furthermore, established quality and safety protocols for phage production are scarce. While pharmaceutical goods are subject to tight restrictions, a few recognized guidelines exist for the research and application of phages [107]. Researchers, patients, and consumers may be unsure about the effectiveness of phages and the ongoing competition between phages and their bacterial hosts [115]. Phages in the gut microbiota are still being researched, with future studies needed to address gaps and existing research methodologies presenting obstacles in understanding viral dark matter.

### 7.4. Limitations of Phage Therapy

#### 7.4.1. Specificity and Resistance Development

One of the main limitations of phage therapy is the high specificity of phages, which can be advantageous for targeted treatment but may also limit their effectiveness against diverse bacterial strains [116]. Phages are highly specific to their bacterial hosts, and a single phage may not be effective against all strains of a particular bacterial species. This specificity necessitates isolating and characterizing multiple phages to cover a broader range of bacterial strains, which can be time-consuming and resource-intensive [117].

Moreover, bacteria can develop resistance to phages through various mechanisms, such as mutations in phage receptors, the production of extracellular matrix, or the acquisition of antiphage defense systems like restriction–modification systems and CRISPR-Cas systems [118, 119]. The development of phage resistance can limit the long-term efficacy of phage therapy. It may require the continuous isolation of new phages or phage cocktails to counteract resistance [120].

#### 7.4.2. Immunogenicity and Safety Concerns

Another challenge associated with phage therapy is the potential immunogenicity of phages. As foreign particles, phages can trigger an immune response in the human body, producing antiphage antibodies [39]. The immune system may recognize and clear phages from the body, reducing their therapeutic efficacy and potentially causing adverse reactions [121]. The immunogenicity of phages can vary depending on factors such as the route of administration, the dosage, and the individual patient's immune status [37].

Furthermore, phage therapy's safety must be thoroughly evaluated before widespread clinical application. While phages are generally considered safe and have been used in various settings without significant adverse effects, there are concerns regarding their potential to transfer virulence factors or antibiotic resistance genes to other bacteria through HGT [122, 123]. Selecting appropriate phages and rigorous screening for undesirable genetic elements is crucial to minimize these risks [124].

#### 7.4.3. Delivery and Stability Issues

Effective delivery of phages to the site of infection is another challenge, particularly in the case of intracellular pathogens like Mtu [125]. Phages must overcome various physical and biological barriers to reach their target bacteria, such as the stomach's acidic environment, the presence of enzymes, and the host immune system [126]. Developing suitable formulations and delivery systems, such as encapsulation or surface modification, may be necessary to protect phages and ensure their stability and efficacy [127].

Moreover, phages may have limited stability under certain environmental conditions, such as extreme temperatures, pH variations, or exposure to UV light [128]. The storage and transportation of phages require careful consideration to maintain their viability and infectivity [129]. Adequate quality control measures and standardization of phage preparations are essential to ensure consistent and reliable therapeutic outcomes [130].

In conclusion, while phage therapy holds promise as an alternative or complementary approach to combat drug-resistant tuberculosis, several limitations and challenges need to be addressed. These include the high specificity of phages, the development of phage resistance, potential immunogenicity and safety concerns, and issues related to delivery and stability. Ongoing research efforts aim to overcome these challenges and optimize phage therapy for clinical application in treating TB and other bacterial infections.

### 7.5. Clinical Application

Amit Kumar Singh's research has shown the ability of the D29 mycobacteriophage LysB protein to effectively destroy drug-resistant strains of Mtu [131]. LysB efficiently breaks down MDR Mtu, either by itself or when combined with anti-TB medications. The researchers found that the surfactant Tween-80 boosts the activity of LysB. The study shows that LysB can eradicate Mtu in mouse macrophages [132]. The results suggest that LysB might be a promising phage-derived treatment and should be tested in preclinical and clinical studies. This study is the first to demonstrate the impact of LysB on drug-sensitive and drug-resistant Mtu strains in both intracellular and extracellular settings. LysB might serve as an adjuvant to enhance the existing antibiotic treatment. Additional study is required to assess its effectiveness in animal models and confirm its safety before integrating it into conventional therapy protocols. This work demonstrates the antimicrobial effectiveness of LysB against Mtu infection and establishes a basis for new treatment strategies [16].

Fan Yang's research discovered that bacteriophage strains D29 and DS6A may efficiently eradicate Mtu H37Rv on 7H10 agar plates. Phage DS6A is the only one that effectively kills H37Rv in liquid culture and human primary macrophages infected with Mtu. Follow-up studies demonstrated that humanized mice infected with aerosolized H37Rv and then treated intravenously with DS6A showed enhanced body weight and better lung function than the control group. DS6A efficiently decreases the Mtu burden in mouse organs, especially in the spleen. The results support the possibility of using phage therapy as a successful treatment for Mtu infection [15].

## 8. Conclusion

The human gut phageome is a complex ecological system that influences the gut microbiota and significantly affects health and illness. The gut phageome's interaction with Mtu sheds light on drug-resistant TB's development and treatment. According to recent research, Mtu survival, pathogenicity, and treatment resistance may be affected by gut phageome interactions such as HGT and host immune response regulation. Phages can transfer antibiotic resistance genes to particular bacterial hosts, possibly helping drug-resistant TB strains grow. Phage therapy, a customized drug-resistant TB treatment, is promising. Current phage therapy research reveals that mycobacteriophages can target and lyse Mtu, an alternative or supplement to antibiotics. To fully employ phage treatment, phage susceptibility, restricted repertoire, and TB pathogenesis complexity must be addressed. Understanding the evolutionary arms race between phages and their bacterial hosts is essential for phage-based therapeutics. Phages and bacteria interact dynamically, and phage resistance can arise, stressing the need for greater research to enhance phage therapy and reduce resistance risk. Finally, the gut phageome may fight drug-resistant TB. Studying the intricate relationships among phages, Mtu, and the human immune system might aid in developing novel drug resistance therapies and enhancing patient prognosis. Further research, clinical trials, and international collaboration are crucial in advancing the use of the gut phageome to combat drug-resistant TB.

## Figures and Tables

**Table 1:** Summary of studies conducted on microbiome/phageome.

Article title	Year	Aim of study	Result/conclusion	Ref
Bacteriophages, gut bacteria, and microbial pathways interplay in cardiometabolic health	2024	How does the phageome impact cardiometabolic function by interacting with gut bacteria and influencing their activities?	The benefits of phage therapy and fecal virome transplant (FVT) extend beyond bacterial infections, showcasing their potential in targeting other diseases involving bacteria or other microorganisms in different organs within the body.	[14]
Bacteriophage therapy for the treatment of *M. tuberculosis* infections in humanized mice	2023	Can phage DS6A effectively target and kill wild-type Mtu H37Rv in laboratory settings, including in vitro conditions, essential human macrophages, and in refined mouse models?	DS6A demonstrates a reduction in Mtu levels within various organs of mice, with a more significant impact observed in the spleen. These results highlight the potential of developing phage therapy as an effective treatment option against Mtu infections.	[15]
Mycobacteriophage D29 lysin B exhibits promising anti-mycobacterial activity against drug-resistant *M. tuberculosis*	2023	Is D29 LysB capable of serving as a viable treatment option to combat *M. tuberculosis*, potentially offering an alternative to conventional antibiotics?	Notably, LysB shows promise as a candidate for enhancing the current anti-infection regimen, particularly when used in conjunction with isoniazid and rifampicin. While the in vitro studies reveal an additional antibacterial effect of LysB when combined with existing medications, further research is needed to confirm its efficacy in animal models. Nevertheless, these findings provide a proof of concept regarding the antimicrobial activity of LysB against *M. tuberculosis*.	[16]
Microbiome-phage interactions in inflammatory bowel disease	2023	Can bacteriophages be utilized as a targeted approach to eliminate pathobionts, to both prevent and treat symptoms and manifestations associated with inflammatory bowel disease (IBD)?	Exploring the interactions between endogenous phages and gut commensals in gastrointestinal tract infections (GTI) and inflammatory bowel disease (IBD) could pave the way for precision microbiome modulation using phages. This approach holds promise for managing IBD and other noncommunicable diseases linked to the microbiome.	[17]
Applications of bacteriophages against intracellular bacteria	2022	How effective are bacteriophages in combating intracellular microbes? What strategies can be employed by phages to effectively target and kill the most common intracellular microorganisms?	There is a notable potential for utilizing genetically engineered phages or phage particles like lysine in combating intracellular microorganisms, particularly targeting pathogens like *M. tuberculosis*.	[18]
Interactions between bacterial and phage communities in natural environments	2022	How does the composition and evolution of phage communities contribute to their functionality in regulating the population and evolutionary dynamics of bacterial communities?	Investigating the interplay between phages and bacteria in natural settings is crucial for understanding their implications on eukaryotic hosts, including how phages interact with the human immune system to eliminate bacterial infections. Such research is vital for the development of phage-based therapies and for expanding our foundational knowledge in this field.	[19]
Mycobacteriophages as potential therapeutic agents against drug-resistant tuberculosis	2021	What are the key characteristics of mycobacteriophages and how do they function in targeting and killing *M. tuberculosis*? What are the advantages and limitations of using them as therapeutic or preventive agents against drug-resistant strains of *M. tuberculosis*?	The utilization of mycobacteriophages for treating drug-resistant tuberculosis relies on understanding their impact on the lung microbiome and host immunity. Implementing selective, safe, and standardized phage preparations in combination with conventional drugs and host immune responses could provide a viable solution for treating otherwise untreatable TB cases. Developing bioinformatics tools to predict phage efficacy against drug-resistant *M. tuberculosis* strains and consider the host tissue environment is essential for identifying effective phage treatments.	[20]
Dynamics of mycobacteriophage mycobacterial host interaction	2020	In what ways can molecular models be utilized to understand how bacteriophages engage in effective battles against *Mycobacterium*, specifically in stimulating immune responses or inhibiting bacterial growth?	In the domain of genetic manipulation, mycobacteriophages have shown potential in enhancing traits in mycobacteria. To leverage bacteriophages as a therapeutic strategy, a comprehensive understanding of their influence on bacterial populations is essential. Utilizing computer simulations can offer valuable insights into specific interactions between mycobacteriophages and their hosts. Simulation models enable a deeper comprehension of system behavior under varying parameters, aiding in the prediction and understanding of phage-host interactions.	[21]

## Data Availability

Data sharing is not applicable to this article as no new data were created or analyzed in this study.
